# The salivary metatranscriptome as an accurate diagnostic indicator of oral cancer

**DOI:** 10.1038/s41525-021-00257-x

**Published:** 2021-12-08

**Authors:** Guruduth Banavar, Oyetunji Ogundijo, Ryan Toma, Sathyapriya Rajagopal, Yen Kai Lim, Kai Tang, Francine Camacho, Pedro J. Torres, Stephanie Gline, Matthew Parks, Liz Kenny, Ally Perlina, Hal Tily, Nevenka Dimitrova, Salomon Amar, Momchilo Vuyisich, Chamindie Punyadeera

**Affiliations:** 1 Viome Research Institute, Viome Life Sciences, Inc., New York City, USA; 2 Viome Research Institute, Viome Life Sciences, Inc., Seattle, USA; 3 The Saliva and Liquid Biopsy Translational Laboratory, Institute of Health and Biomedical Innovation, Queensland University of Technology, Brisbane, QLD 4059 Australia; 4 The Translational Research Institute, Woolloongabba, Brisbane, QLD Australia; 5 The School of Medicine, University of Queensland, Royal Brisbane and Women’s Hospital, Brisbane, QLD Australia; 6 New York Medical College, Valhalla, NY USA

**Keywords:** Oral cancer detection, Bacterial genes, Diagnostic markers, Data processing

## Abstract

Despite advances in cancer treatment, the 5-year mortality rate for oral cancers (OC) is 40%, mainly due to the lack of early diagnostics. To advance early diagnostics for high-risk and average-risk populations, we developed and evaluated machine-learning (ML) classifiers using metatranscriptomic data from saliva samples (*n* = 433) collected from oral premalignant disorders (OPMD), OC patients (*n* = 71) and normal controls (*n* = 171). Our diagnostic classifiers yielded a receiver operating characteristics (ROC) area under the curve (AUC) up to 0.9, sensitivity up to 83% (92.3% for stage 1 cancer) and specificity up to 97.9%. Our metatranscriptomic signature incorporates both taxonomic and functional microbiome features, and reveals a number of taxa and functional pathways associated with OC. We demonstrate the potential clinical utility of an AI/ML model for diagnosing OC early, opening a new era of non-invasive diagnostics, enabling early intervention and improved patient outcomes.

## Introduction

Oral cancer (OC) is a major subtype of head and neck cancers (HNC)^1^. Worldwide, there are an estimated 350,000 to 400,000 new cases of OC each year, and more than 150,000 deaths^2^. In the USA in 2020, it is estimated that 53,500 people (~71% male) will be newly diagnosed, and that there will be 10,860 deaths (~73% male) from OC. That amounts to 145 new cases diagnosed every day, and one person dying from OC every hour. The overall 5-year survival rate for people with OC is 40% and this figure has not improved in the past 40 years, resulting in more cancer deaths when compared to melanoma and cervical cancer in the USA^3^. However, if diagnosed at an early stage, the overall 5-year survival rate is 84%. Unfortunately, with today’s practices, only 29% of patients are diagnosed at an early stage.

The cost effectiveness of targeted screening/early diagnostic approaches (these terms, as well as “early detection” are used interchangeably throughout this paper) has been supported by the results from a simulation model study^4^. Currently, OC is hard to detect in the early stages because of the lack of effective early diagnostic tools, resulting in late diagnosis, leading to poor prognosis and low survival rates^5,6^, with a significant impact on the healthcare system. Major risk factors for the development of OC are excessive tobacco smoking, alcohol consumption, and in Asia, betel nut chewing. Tobacco use can include consuming tobacco products by smoking, chewing, vaping, etc. OC risk increases with age or a history of tobacco use^7,8^, and the increase becomes more rapid after 50 years of age^9^. Only 2–4% of OC cases are associated with human papillomavirus (HPV) infection. In addition, OC commonly occurs in people without a history of tobacco use or alcohol consumption, which argues that additional environmental factors may lead to the development of OC.

Existing microbiological literature has established a significant correlation between changes in the microbiome and cancer phenotypes^10,11^. Perhaps the best-known association is of bacteria (*Helicobacter pylori*) causing gastric ulcers that progress into gastric cancer. In the last decade, multiple microbiome studies using biopsies, tissue samples, and deep epithelial swabs taken from OC patients have shown associations of certain microbes with the development of OC. In previous studies, although there were significant methodological variations in terms of type of samples, technologies used for microbial analysis (16S rRNA gene sequencing or shotgun DNA analysis), design and inclusion criteria, some overlaps were observed at high taxonomic levels. More recently, the notion has emerged that the microbial association with OC is at the level of the microbial community’s function, rather than at its composition^12^. Most intriguing, recent evidence raises the possibility that changes in salivary microbiome composition may have potential as biomarkers for detecting HNCs^13–17^.

Visual and tactile screening, followed by laboratory testing and clinical assessment remain the backbone of the current clinical standard of care. A simpler alternative would be measurements made from saliva samples. Saliva specimen collection is non-invasive, straightforward, safe, painless; patients can collect samples themselves. As with home-based stool sample collection, we imagine that removing the need for professional healthcare personnel for sample collection could lead to greater potential for access as well as patient compliance compared to blood-based methods^18^. Saliva is also a more stable and a less complex matrix compared to blood and as such, is ideal for broad use^19^. Despite all of these advantages of the use of saliva, an accurate method of profiling the microbiome changes in saliva samples as an early diagnostic indicator has not been developed to date that could generate the much needed clinical impact in this prevalent and deadly disease.

Our overarching aim is to develop a simple, non-invasive, and scalable method, with a classification algorithm that can be used as an early diagnostic tool to address an urgent unmet clinical need. We hypothesized that combining salivary microbial transcriptome (metatranscriptome) profiling using next-generation sequencing (NGS) technology with machine learning (AI/ML) would allow us to develop a classifier that could accurately discriminate premalignant/OC cases from normal healthy controls. We have developed and validated both state-of-the-art techniques for achieving accuracy and robustness in our OC classifier: (1) NGS metatranscriptomic analysis, which captures the microbial activity (RNA) within the saliva sample in high resolution, and accurately identifies both the microbial taxonomies as well as the microbial functions^20^, and (2) analytical discovery of the metatranscriptomic signature associated with OC, using a model trained from a ML algorithm.

To achieve the above objectives, we collected 433 saliva samples and meta-data from 242 unique individuals, and divided these samples into the cohorts described in Table 1 below. Using these cohorts, we developed and evaluated classifiers for two scenarios:

1. Screening for OC or oral premalignant disorders (OPMD) within the high-risk population, i.e., 50 years or older, OR with a history of tobacco use
2. Screening for OC only within the average-risk population, i.e., general population across all backgrounds

**Table 1:** Study cohorts.

	A: High-risk OC+OPMD discovery cohort	B: High-risk OC+OPMD cross-validation (A+27 samples)	C: Average-risk OC-only (OC subset of A + 7 average-risk)	D: Average-risk technical validation	Total unique across all cohorts
Number of participants	117	144	99	91	242
Controls	59	75	49	91	171
Cases	58	69	50	n/a	71
Number of samples total	117	117 from Cohort A+	92 from Cohort A+	282	n/a
Unique samples	117	27	7	282	433
Cases	58	69	50	n/a	71
Pre-malignant	10	14	n/a	n/a	14
Malignant	48	55	50	n/a	57
Sex (% female)	37.6	37.5	40.4	38.7	38.8
Controls	54.2	50.7	57.1	38.7	
Cases	20.7	23.2	24	n/a	
Age (y) mean ± std	60.2 ± 11.3	61.4 ± 11.4	59.7 ± 12.6	22.6 ± 10.5	37.2 ± 21.7
Controls	56.3 ± 10	58.5 ± 11	56 ± 10.8	22.6 ± 10.5	
Cases	64.1 ± 11.4	64.5 ± 11.1	63.3 ± 13.3	n/a	

The 433 unique samples in this study (57 OC samples, 14 OPMD samples, and 362 cancer-free samples) are organized into 4 cohorts A, B, C, and D according to the study goals. High-risk population is 50 years or older OR a history of smoking (current or past smoker). Average-risk population is the general population across all backgrounds and histories.

While we provide the results for both scenarios, we highlight the high-risk OC + OPMD screening scenario in the rest of the paper, since this represents the largest unmet clinical need. Based on our analysis and results across cohorts, the findings from this study provide the foundation for a large multi-center clinical trial to validate the effectiveness of the diagnostic classifier on the populations of interest.

## Results

### Cohort description

The goal of this study was to evaluate diagnostic performance of a novel liquid biopsy on both a high-risk as well as an average-risk population. Table 1 summarizes the participants in the cohorts used in this study: Cohorts A and B represent the *high-risk population*, defined as people aged 50 years or older OR with a history of tobacco use (so a 55 year old never-smoker and a 25 year old smoker would both belong to these cohorts). Cohorts C and D represent the average-risk general population.

**Fig. 1:**
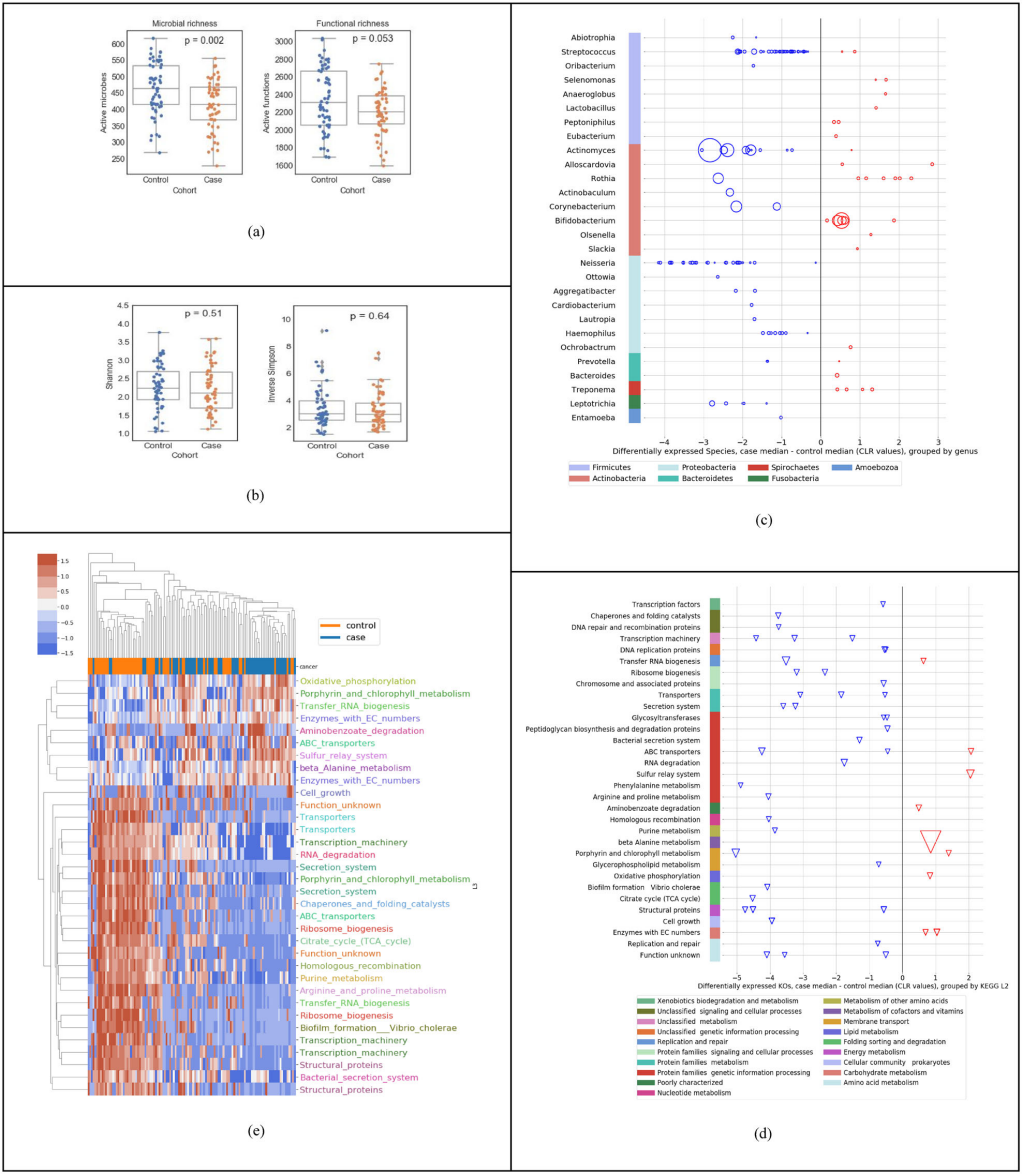
Descriptive statistics of salivary metatranscriptome of the high-risk population (Cohort A in Table 1). **a** Species richness; control median 463, case median 415 and function richness; control median 2306, case median 2205. **b** Shannon diversity index; control mean 2.25, case mean 2.20; and Inverse Simpson diversity index; control mean 3.41, case mean 3.26. **c** Using Mann–Whitney *U* tests and at least twofold difference in means (0.69 in CLR space), 139 differentially expressed species (at *p* < 0.05) up- or downregulated (red and blue respectively) in cases relative to controls, organized by genus and phylum (median difference in CLR values); the size of the bubble is inversely proportional to the *p* value. **d** Using Mann–Whitney *U* tests and at least twofold difference in means (0.69 in CLR space), 49 differentially expressed KOs (at *p* < 0.05) up- or downregulated in cases relative to controls, organized by KEGG level-3 and level-2 functional groups; the size of each triangle is inversely proportional to its *p* value **e** Clustermap using Euclidean distance of CLR transformed sum(transcripts per million) data for active function (KO) features significant by Mann–Whitney *U* tests. Features are shown with corrected *p* values < 0.01 and median CLR differences between the cohorts of greater than 0 or less than −1. KOs are color coded by their KEGG level-3 functional group.

**Fig. 2:**
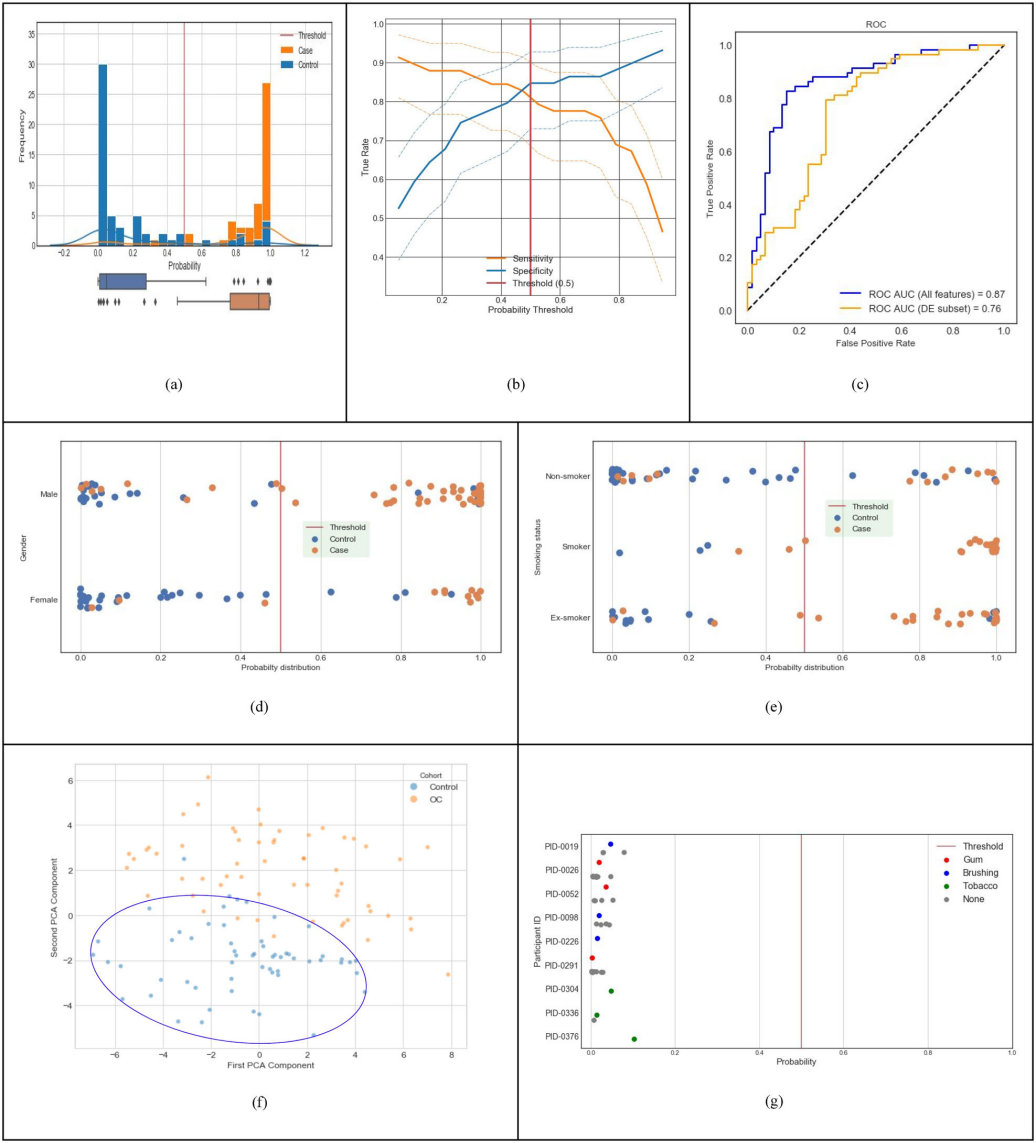
Predictive performance of machine-learnt classifier trained with discovery dataset (Cohort A in Table 1). **a** Distribution of classifier output probabilities across the sample set. **b** Sensitivity & specificity tradeoff with 95% confidence interval computed using the Clopper-Pearson method; at the default decision boundary of 0.5, sensitivity is 0.81 and specificity is 0.85. **c** ROC AUC of the classifier using the LOOCV method is 0.87 (blue curve); using differentially expressed features only is 0.76 (orange curve). **d** Classifier probabilities separated by gender. **e** Classifier probabilities separated by smoking status. **f** PCA analysis using top 100 features (PC1 and PC2 capture 10.2% and 6.3% of the total variation, respectively.). **g** Probability of cancer output from the classifier for control samples with and without interference from chewing gum, chewing tobacco, and brushing teeth.

**Fig. 3:**
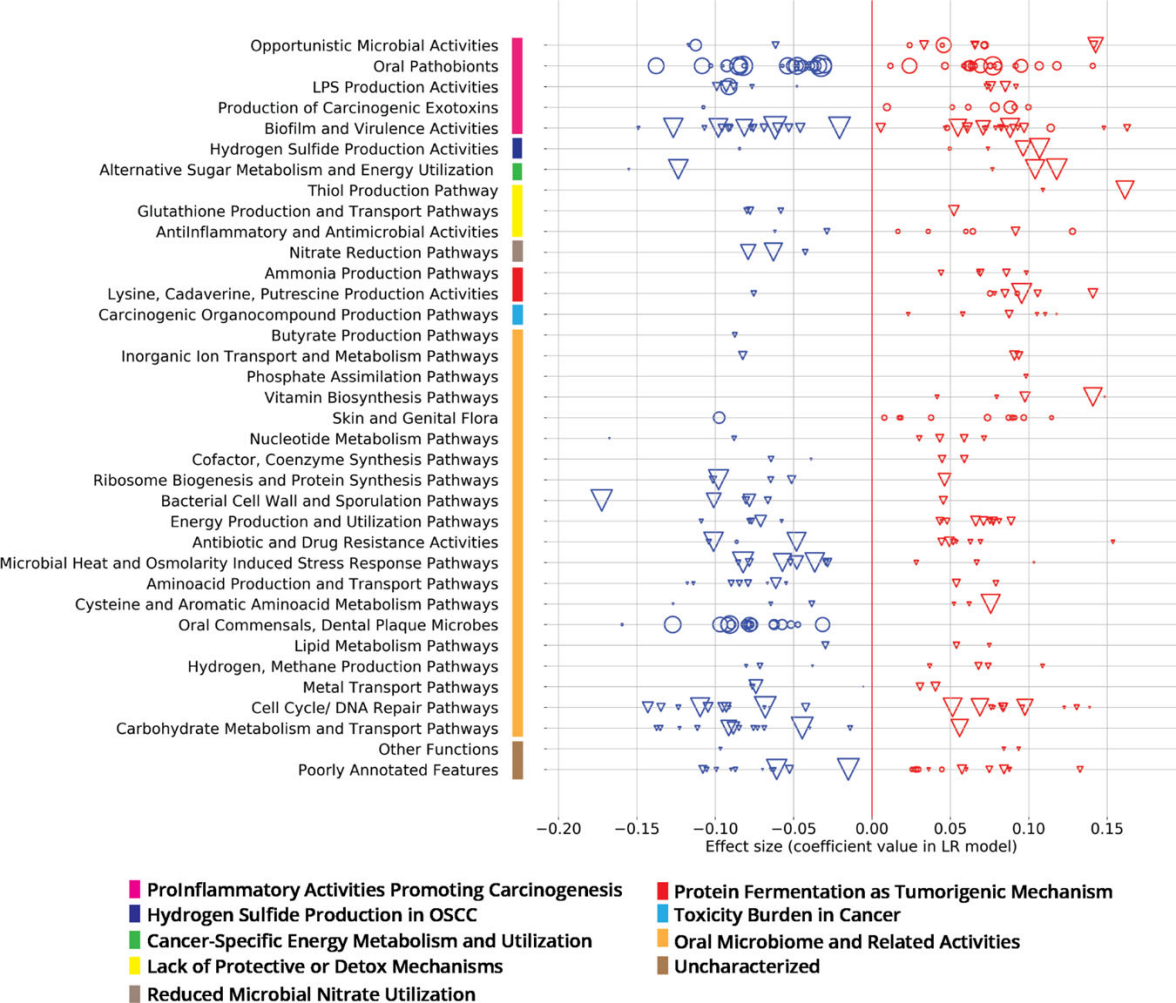
Oral metatranscriptomic signature from the ML classifier trained with Cohort A from Table 1. Effect sizes (coefficient values within the classification model) of 101 active species (circles) and 247 active KOs (triangles), grouped into curated Viome Functional Categories (VFC), see ‘Supplementary Note 4’ section of the Supplementary Material; sizes of circles or triangles are proportional to the CLR median difference in expression level between cases and controls.
- The goal of Cohort B (high-risk cross-validation cohort) was to evaluate the performance of our approach by including an additional 27 samples on top of Cohort A.
- The goal of Cohort C (average-risk OC only) was to develop and evaluate a classifier for a broad general population, and with only OC cases (i.e., without the premalignant OPMD cases).
- The goal of Cohort D was to perform a technical validation using samples from “presumed normal” individuals from the general population, and to evaluate whether external interference factors (such as chewing gum, chewing tobacco, or brushing teeth) influenced the metatranscriptomic analysis.

- The goal of Cohort A (high-risk OC + OPMD discovery cohort) was to support the primary use case of this study—to develop a machine-learned classifier for early diagnosis in the high-risk cohort, to analyze the features in the raw data (Fig. 1), evaluate the classifier performance (Fig. 2 and Table 2), and summarize the metatranscriptomic signature (Fig. 3). For this objective, we included both OC and OPMD patients within the positive “cases” category, as one would expect in a clinical early detection or screening test.

**Table 2:** Model performance for cohorts described in Table 1.

	A: High-risk OC + OPMD	B: High-risk CV OC + OPMD	C: Average-risk OC only	D: Average-risk Technical validation
ROC AUC	0.87	0.87	0.90	n/a
Sensitivity	81%	83%	76%	n/a
Specificity	85%	79%	88%	97.9%
True positives by stage				
OPMD	7/10	11/14	n/a	n/a
OC Stage 1	12/13	11/14	12/14	n/a
OC Stage 2	11/16	12/17	11/16	n/a
OC Stage 3	1/2	2/2	2/3	n/a
OC Stage 4	13/14	18/19	10/14	n/a

For sensitivity and specificity, we used the standard default clinical decision threshold of prediction probability = 0.5. Technical validation for the average-risk cohort D was performed using the model developed for Cohort A.

### Descriptive statistics

Figure 1 summarizes a set of descriptive statistics to show the differences in active species and KOs between the 58 cases and 59 controls in our study. Across all samples used in this study, we detected a wide range of unique active microbes (1587 active species, sample mean 438 ± StD 81) and unique active functions (4932 KEGG Orthologs or KOs, sample mean 2270 ± 314). As shown in Fig. 1a, we observed a lower richness in cases compared to controls, both in terms of active species and active KOs. However, as shown in Fig. 1b, we do not see a statistically significant difference (using Mann–Whitney *U* (MWU) test) in diversity indices, such as the shannon index or the inverse simpson diversity index between cases and controls. In general, diversity measures both richness and evenness, and in this dataset, although there is a lower richness in active species, Pielou’s evenness index is not significantly different between cases and controls (*p* value = 0.72). Overall, the ecological change in decreasing richness in cases does not seem to be driven by a single dominating species. Figure 1c shows the up/down regulation of the 139 significantly differentially active species between cases and controls, grouped into 28 genera (*p* < 0.05, MWU test). Each dot (circle) represents a species, all species on a horizontal line are grouped by the genus on the left of the line, and all genera in a given color are grouped by the phylum shown at the bottom. We observed a downward shift, i.e., that 75.5% of the species are downregulated in cases compared to controls. For example, out of the 41 differentially active species from the *Streptococcus* genus, 39 were downregulated, most of them within 2 units on the centered log-ratio (CLR) scale; the 27 species from the *Neisseria* genus were all downregulated in cases, with many of them at 4 units on the CLR scale. In contrast, 6 species from the *Rothia* genus were upregulated in cases compared to controls. Figure 1d shows the up/down regulation of the 49 significantly differentially expressed KOs between cases and controls, grouped into 30+ KEGG level-3 functional groups (*p* < 0.05, MWU test). Each dot (triangle) represents a KO, all KO’s on a horizontal line are grouped by the “level-3 KEGG function” on the left of the line, and all level-3 KEGG functions in a given color are grouped by the level-2 KEGG function shown at the bottom. We observed that most of the microbial functions were downregulated in cases (81.6%), compared to controls. Finally, Fig. 1e shows a visible distinction between cases and controls using only the differential expression of functions.

It is important to note that Fig. 1 presents a *descriptive* statistical analysis of the 58 cases versus 59 normal controls. The differential expression of individual features taken one-at-a-time without interactions provides a level of insight into the raw data, but may not necessarily result in the highest performing diagnostic model. In the section below, we demonstrate that a linear regression machine-learning approach provides a significantly higher diagnostic performance, as shown in Fig. 2c.

### Predictive performance of the machine-learned (ML) classifier

Figure 2 depicts the clinical diagnostic performance of our trained classifier within the discovery dataset (Cohort A, *n* = 117 in Table 1), using the leave one out cross validation (LOOCV) method described earlier. For each incoming validation sample, the trained model outputs a probability that the input sample belongs to the OC/OPMD class (cases). When this probability is above the clinical decision threshold of 0.5, the sample is classified as OC/OPMD (case), otherwise Not-OC/OPMD (control). We used the default probability value of 0.5 for the clinical decision threshold, since it minimizes loss on the training data, and has the advantage that it balances sensitivity and specificity in general. Figure 2a shows the probabilities output by our model for all samples in cross validation. Our classifier results are bimodal with good separation of cases and controls, and most data points have predicted probability close to 0 or 1 with very few near the clinical decision threshold. The sensitivity and specificity tradeoff with 95% confidence interval is shown in Fig. 2b. At the clinical decision threshold of 0.5, the sensitivity is 0.81 and specificity is 0.85. Finally, Fig. 2c shows that our classifier has an ROC AUC of 0.87. Note that a classifier constructed using only the differentially expressed features shown in Fig. 1 (139 taxa and 49 KOs) performs at ROC AUC of 0.76 (shown by the orange line in Fig. 2c).

Figure 2d illustrates that gender does not overly bias our classifier. Figure 2e shows that smoking history also does not bias our classifier. It detects non-smokers who have cancer, and it detects smokers and ex-smokers who do not have cancer. Figure. 2f shows PCA clustering analysis of samples using the top 100 model features, which shows that non-cancer samples are clustered together, providing evidence that the signature is relatively stable. In addition, nine volunteers (from Cohort D in Table 1) provided saliva samples with different potential interferants, such as chewing gum, chewing tobacco, and brushing teeth. Fig. 2g shows that the probability output of the classifier does not change based on the presence of an interfering substance, showing that our cancer classifier is robust. Taken together, this data demonstrates that our model’s performance and robustness is state of the art in the field^16,21^.

Table 2 gives a summary of the classifier performance for all cohorts in Table 1. The larger high-risk cross-validation Cohort B resulted in a similar performance as Cohort A. The cross-validation performance for a model trained with Cohort C is higher than Cohorts A & B since Cohort C consists of only OC cases without any of the OPMD cases. Cohort D was evaluated with the diagnostic model developed for the primary use case presented in this paper for Cohort A (i.e., the model was trained only with Cohort A, but evaluated with samples from Cohort D). The purpose was to ensure that this model is still able to correctly classify a general population, which was confirmed with a specificity of 97.9% (276 true negatives and 6 false positives). Additional details are in the ‘Supplementary Note 3’ section of Supplementary Material.

### Metatranscriptomic signature from the ML classifier

Figure 3 depicts the details of the features that drive our predictive model. As described earlier, our “metatranscriptomic signature” consists of 348 features (101 active species and 247 active functions) from the intersection of models built in each fold of cross validation. Here, we introduce a curated set of pathway and taxa categories called ‘Viome Functional Categories’ (VFCs) that group all the features into 9 major biological themes comprising 36 functional categories. These VFCs shed light on some of the microbial activities and biological pathway mechanisms that are known to be associated with oral carcinogenesis. For example, the functional category “Opportunistic Microbial Activities” consists of 3 features (1 taxon and 2 KOs) with a negative effect in the classifier, and 9 features (5 KOs and 4 taxa) with a positive effect. A brief description of the VFCs, the themes and the features (taxa and KOs) constituting the themes are provided in ‘Supplementary Note 4’ section of the Supplementary Material.

The functional categories and features within ‘ProInflammatory Activities promoting Carcinogenesis’, ‘Hydrogen Sulfide Production’, ‘Cancer-specific Energy Metabolism and Utilization’, ‘Lack of Protective or Detox Mechanisms’, ‘Reduced Microbial Nitrate Utilization’, ‘Protein Fermentation’, and ‘Toxicity Burden’, are more direct in terms of their association in oral carcinogenesis. This can be seen by the presence of a greater number of features (taxa and KO) that have a positive effect from the model in the ‘Opportunistic Microbial Activities’ and ‘Hydrogen Sulfide Production’ and ‘Production of Carcinogenic Exotoxins’ themes. These themes have already been implied in oral carcinogenesis^22,23^. Amongst the other functional categories, the features are more associated with general Oral Microbiome related Activities and are more predictive of controls. The ‘Oral Commensals, Dental Plaque Microbes’ constitute microbes such as commensal *Streptococcus* sp. that are important in maintaining oral commensalism and microbiome balance^24^. The functional categories that harbor many features have a negative effect from the model include pathways supporting normal cellular metabolism such as ‘Carbohydrate Metabolism and Transport Pathways’, ‘Amino Acid Production and Transport Pathways’, ‘Microbial Heat and Osmolarity Induced Stress Pathways’ and themes involved in cell growth, such as ‘Ribosome Biogenesis’ and ‘Cell Wall and Sporulation’.

## Discussion

The 5-year overall survival rate for all OC in the USA is 84% but drops to 39–65% when diagnosed at an advanced stage (percentage dependent on location and extent of metastasis)^25^. Visual and tactile screenings are the foundation of the current standard of care, usually performed by dental hygienists and primary care physicians, which while being quick and easy, are subjective (e.g., verbal questions about symptoms) and prone to a high number of false negatives and false positives^26^. Research has shown some of the reasons for late diagnosis, which is a layered and complicated problem, including under-utilization of dental and primary care, lack of and poor quality of screening in individuals at a higher risk of developing OC and who do not seek general care, and especially, the fact that in the earliest, most treatable stages, many OC show few symptoms and may not be visible^26–31^.

To get an approximation of the performance of the standard of care, we used data from a meta-analysis of studies used in the clinical assessment of OC^32^ using the standard of care techniques described above (i.e., visual/tactile examinations). Walsh et al. included ten studies in their review along with the assessment methodology, sensitivity, specificity, population size and location for each. We focused on studies that best reflected our sample populations, i.e., predominantly white populations with a mix of all other ethnicities. This resulted in four studies ([‘julien_95a’, ‘downer’, ‘julien_95’, ‘sweeny’]) from the UK and the USA with a combined total of ~2500 participants. We then took a weighted average for sensitivity across the four relevant studies, weighing the metrics by study population size. This yielded a sensitivity value of 0.717 and specificity of 0.989. The diagnostic system presented in this paper yields a sensitivity of 83% for our validation Cohort B, and a specificity of 97.9% for our largest sample set of 282 samples in Cohort D. This establishes that the work presented in this paper is superior to the current standard of care, and we continue to improve this performance with ongoing studies.

Several adjunct diagnostic tools are available to aid providers in identification and diagnosis of OCs^33^, such as brush cytology^34,35^, toluidine blue staining^36^, and light-based visual detection systems^37^. The use of these tools varies among providers, and currently, none of the available tools has been studied sufficiently to prove that their use improves the sensitivity and specificity of the current standard of care physical exam^38,39^. The work presented in this paper addresses these issues in the standard of care. We introduce a method which has non-invasive and easy sample collection using saliva rinse, coupled with an objective and robust classification algorithm with high sensitivity and specificity to distinguish between control samples and OC samples.

There has been interest in investigating either individual bacteria or shifts in microbiome composition and their potential association with different stages of cancer development, since the classification of *Helicobacter pylori* as a causative agent for stomach cancers. In addition, there have been many published studies on the potential association between changes in the microbiome (mainly at the metagenomics level) and cancer. Even though microorganisms have been implicated in 15.4% of human malignancies, there is a dearth of knowledge regarding the role of bacteria in the development and progression of OC. Conventional differential expression analysis reported by existing studies^40^ shows statistical differences in microbial features between cases and controls in cancer tissue, but no study has yet presented a microbiome-based predictive classifier using a non-invasive saliva-based sampling method. Furthermore, while the majority of microbiome studies to date have focused on microbial taxonomy (due mostly to the limitation of DNA sequencing), we used a combined taxonomic and functional analysis (metatranscriptomics) and demonstrate that microbial functions make important contributions to our model. This is not unexpected, since the biological activity (of mechanistic relevance to OC biology) is the result of active gene expression, and not just genetic potential encoded by DNA.

In this study, we have used both taxonomic profiling and functional profiling to develop a diagnostic classifier based on AI/ML using salivary metatranscriptomic data. We have detected a wider range of unique active microbes (1587 active species, sample mean 438 StD 81) and unique active functions (4932 KOs, sample mean 2270 ± 314) than previous studies, making it feasible to comprehensively profile bacterial functions (KOs). Our AI/ML diagnostic classifier is effective in identifying individuals who are at high risk of developing OC, starting with premalignant lesions/OPMD (Cohort A in Table 1), which is the largest unmet clinical need in this space. For this cohort, cross validation of our diagnostic classifier yielded an ROC AUC of 0.87, sensitivity of 0.81 and specificity of 0.85. For a narrower use case such as Cohort C which includes only OC cases, our ML model achieves ROC AUC over 0.9. A secondary technical validation using 91 healthy individuals (Cohort D) yielded a sensitivity of 97.9%. To the best of our knowledge, our classifier has the best diagnostic performance published currently.

We have observed a lower richness, both in terms of active species and active KOs in saliva samples analyzed from cases compared to controls (Fig. 1a), corroborating with a previous study by ref. ^40^ using salivary metagenomic analysis. In contrast, another study revealed much greater diversity of bacterial communities in OC samples^41^. Our study shows that several genera such as *Streptococcus*, *Haemophilus*, and *Actinomyces* downregulated as does Yang’s work^42^; although some genera like *Fusobacterium* does not appear to be differentially expressed in our analysis. Our high throughput metatranscriptomic technology can detect features (strain-level taxa as well as KOs for functional activity) at a much finer granularity compared with 16S techniques used in Yang’s work^20,43^. Nevertheless, this level of concordance with prior work is highly encouraging. We have also detected at the genus-level high amounts of periodontal bacteria *Fusobacterium*, *Prevotella* and *Porphyromonas* in saliva samples from OC and OPMD, confirming previous findings^44^. Furthermore, we believe that our model is specific to OC and does not overlap with other common conditions such as canker sores, since there is negligible overlap (two species) between the features of our signature and the microbial signature discovered by Kim^45^.

Among the ProInflammatory Activities promoting carcinogenesis, we identified several species of pathobionts from *Porphyromonas*, *Treponema*, *Fusobacterium*, and *Streptococcus* genera and their raffinose, stachyose, and melibiose transporters, as previously reported^46–48^. This theme also captured two *Porphyromonas* species and one microbial KO shown to produce proinflammatory mediators^49,50^ and eight KOs that are involved in biofilm formation and virulence^51,52^. Protein Fermentation and polyamine metabolism are known to be associated with tumorigenesis by mediating oxidative damage to the host cells^53^, we report protein fermentation and ammonia-producing KOs as predictors of OC^54–56^. Five toxin-generating KOs that produce benzaldehyde, arsenite, and other carcinogenic metabolites also contribute to the pathogenesis of OC^22,23,57^.

Species-level taxonomic classifications were essential for identifying relevant taxa that are predictive of the phenotype. This is clearly depicted in Figs. 1c and 3, where several genera contain multiple species and that make opposite contributions to the model. This is an important observation, as there are many literature reports that show genera as contributing to a phenotype. In reality, that finding may be driven by certain species within the genera, but other species may have the opposite effect. Therefore, genus-level analysis can lead to false results of a test, depending on the specific species present in a sample.

Our approach improves on previous functional methods by revealing not simply differential expression and functional categorization, but more importantly, mechanisms that integratively connect predictive gene-encoded active functions along with active microbes to relevant biological themes characteristic of OC. Understanding the systems biology level perspective revealed by our ML model can take us one step closer to developing not only diagnostic but also future therapeutic strategies to address this disease.

Ideally, the diagnostic classifier developed in this study would be used clinically as an early detection/screening tool for a high-risk population (adults of either sex 50 years or older OR those with a history of tobacco use). A positive result may indicate the presence of either OPMD or OC and should be followed by, for instance, a detailed physical examination and/or a biopsy by an appropriate medical practitioner (dental surgeon, ENT specialist, etc). Due to the simple, efficient and non-invasive nature of the saliva collection procedure, it is unlikely that such a prediction model will cause any potential adverse effects. The primary risk associated with this prediction model is the possibility of a false prediction (i.e., a false-positive or a false-negative result). All positive test results will need to be followed by a physical examination of the patient. In a situation where the system presented here produces a false-negative result, there is a chance that a case of OC could go undetected, but this risk is no greater than what exists under the current standard of care (visual/tactile examination by a medical practitioner).

The main contribution of this paper is a diagnostic system that addresses an unmet clinical need for early detection of OC (including premalignant cases) in high-risk populations (people 50 years or older OR with a history of tobacco use). Our system uses (a) a simple, non-invasive, saliva sample (b) high throughput NGS metatranscriptomic lab analysis, and (c) a machine-learned diagnostic classifier that accurately discriminates between cases and controls. We show that this system can identify high-risk OPMD/OC patients vs. normal healthy controls with ROC AUC of 0.87. When restricted only to OC patients at average risk, our classifier achieves ROC AUC over 0.9. We demonstrate a system that effectively improves upon the current standard of care globally, opening a new era of non-invasive diagnostics, enabling early intervention and improving patient outcomes.

Our method is based on extracting high-resolution metatranscriptomic (RNA) functional and taxonomic features from saliva samples (rather than genus-level 16S or metagenomic/DNA features), which represents gene expression of active microbial functions in the sample. Second, rather than performing a differential expression analysis of each feature as in most current literature, we perform a machine-learning analysis that captures the inter-dependencies among the thousands of features within the processes and allows us to *predict* the probability of a cancer signature in a sample. This allows us to identify and connect the most important predictive features that represent active microbial functions along with active microbes to relevant biological themes characteristic of OC. While the results in this discovery study are encouraging, and the method used extracts a meaningful signal with reduced overfitting, we recognize the limitations of the number of samples in the current study, and plan to perform a large multi-site study to validate the signature on a broader scale.

Overall, we believe that the AI/ML-based diagnostic classifier developed and validated in this study opens a new era of non-invasive diagnostics, enabling early intervention and improving patient outcomes, while significantly reducing healthcare costs. Once an early diagnostic test is available at scale, we can routinely improve the accuracy of our test as we collect more “real-world evidence” to further train our machine-learning models. This enables *de novo* discoveries that will have a great impact and open a new era of precision medicine.

## Methods

### Study cohorts

For Cohorts A, B, and C, we recruited 71 newly diagnosed treatment-naive patients with OC and OPMD, and collected a saliva sample from each of them at baseline. In addition, we collected 362 saliva samples from 171 non-diseased individuals across all cohorts shown. The exact inclusion and exclusion criteria are described in the ‘Supplementary Note 1’ section of Supplementary Material. Based on histopathological reports, the clinical stages of patients with OC were classified based on the cancer staging system of the American Joint Committee on Cancer^58^. All patients in Cohorts A, B, and C were HPV negative based on a PCR-based test of their saliva^59^.

This study was approved by the Queensland University of Technology and University of Queensland Medical Ethical Institutional Boards (HREC no.: 1400000617 and HREC no.: 2017000662 respectively) and the Royal Brisbane and Women’s Hospital (HREC no.: HREC/12/QPAH/381) Ethics Review Board. Written informed consent was obtained from all participants and all of the methods in this study were performed in accordance with the relevant guidelines and regulations.

### Sample collection and laboratory analysis

Laboratory analysis of the saliva samples was similar to the metatranscriptomic method designed for large-scale population analysis of stool samples as described previously^20^ (summarized in Supplementary Fig. 2 in the Supplementary Material) and included sample collection, ambient temperature sample preservation, total RNA extraction, physical removal of ribosomal RNAs, preparation of directional Illumina libraries, and Illumina sequencing. The stability of the RNA stabilizer was tested for up to 28 days at ambient temperature, including shipping. (More details in Supplementary Figs. 2 and 3 in Supplementary Material).

### Bioinformatics processing

Paired-end reads were mapped to a catalog of 53,660 microbial genome assemblies spanning archaea, bacteria, fungi, protozoa, and viruses. (We downloaded the complete genomes available in NCBI Reference Sequence Database and used the GenBank sequence database for viral genomes.) Strain-level relative activities were computed from mapped reads via the expectation-maximization (EM) algorithm^60^. Relative activities at other levels of the taxonomic tree were then computed by aggregation according to the taxonomic rank. Relative activities for biological functions were computed by mapping paired-end reads to a catalog of 52,324,420 microbial genes, quantifying gene-level relative activities with the EM algorithm, and then aggregating gene-level activity by KO annotation^61^. The identified and quantified active microbial species and KOs for each sample were then provided to the OC classifier. (More details are in the ‘Supplementary Note 2’ section of Supplementary Material).

### Descriptive statistical analysis

Standard statistical analyses described below were initially performed to analyze the differential expression of active microbes and active functions between the 58 cases and the 59 healthy controls in Cohort A (Fig. 1). The data were transformed using the CLR transformation^62^ after imputation of zero values using multiplicative replacement^63^. We used the two-sided MWU test (*p* < 0.05 after Benjamini–Hochberg correction for multiple comparisons) and at least twofold difference in means (0.69 in CLR space). It is important to note that this is a descriptive statistical test to analyze features independently for differential expression without taking into account the interactions among features and is thus not suitable for the machine-learning classification method (below).

### Mapping KOs to functional categories for presentation

For Fig. 1, the Python module “Bio.KEGG” was used to take as input the KO name and return KO hierarchy at three different levels (level-1 to level-3). For Fig. 3, VFCs, each KO and taxa feature from the ML model was analyzed in the context of expert-assessed directional pathway mechanisms or biologically characterized taxonomic microbial groups (see ‘Supplementary Note 4’ section of Supplementary Material). Subsequently, the VFCs were summarized into broader biological themes based on literature and their relevance to carcinogenesis or OC progression as described in the Discussion and the ‘Supplementary Note 4’ section of Supplementary Material.

### Machine-learning (ML) classifier development and cross-validation

The OC binary classifier was trained using the appropriate number of samples from the population in the cohorts described in Table 1. Each sample was annotated as a case (OC or OPMD) or control. The molecular data (microbial species and KOs) derived from the metatranscriptomic analysis of the saliva samples were used as input features for training. For this study, we chose a logistic regression (LR) model since it performs well and is easily interpretable. In particular, we used *l*_2_ regularized LR with a regularization parameter of 1, implemented in scikit-learn^64^. This choice was motivated by low model complexity as protection against overfitting.

We used “LOOCV” to validate both feature selection and model performance. It is conventionally held that in k-fold validation, as k approaches N (i.e., approaches LOOCV), estimator variance decreases due to increasing number of observations and aggregation over a greater number of folds but increases due to increasing nonindependence of the data comprising each fold (e.g.^65^). Due to the small sample size relative to the number of active microbes and functions, we took precautions to ensure that the features we present are robust to random variation in the data. The following procedure was used:

- To begin with, the features in our molecular data consist of all detected active microbes (1587 species) and functions (4932 KOs).
- Data were transformed using the CLR method^62^. Features with variance less than 25th percentile of the variances of all features were removed as part of data pre-processing and 533 active microbes and 2216 active functions were used for the remaining analysis.
- For each fold of the LOOCV method, we performed feature selection as follows. Bootstrap sampling of each training set 1000 times provided the sampling distribution of all LR coefficients. We considered features where the 95% CI of this distribution did not cross zero to be significant at *p* < 0.05, and used these to estimate the model in each iteration of the LOOCV procedure.
- To obtain a final model for the purposes of follow-on validation or clinical use, we fit an LR model with the 348 features (101 active species and 247 KOs) at the *intersection* of the models built in each fold of cross validation. This is a conservative choice made to select the features consistently selected across cross validation, and therefore reduce overfitting. We call these 348 features used in the final ML model the *metatranscriptomic signature* of OC.

### Reporting summary

Further information on research design is available in the Nature Research Reporting Summary linked to this article.

## Supplementary information

Supplementary Information

Reporting Summary

## Data Availability

This research was sponsored by Viome and the authors of the paper who have access to the data are employees or scientific collaborators of Viome who have signed contracts with Viome to be bound by Viome’s privacy policy and access restrictions. The sample data and feature matrix for the main discovery cohort (Cohort A) has been made available at figshare, at 10.6084/m9.figshare.13244243. Additional data can be made available through a Data Transfer Agreement that protects the privacy of participants’ data; interested researchers may request at https://www.viome.com/vri/data-access. The information provided by interested researchers on the dataset request form will be used to generate a Statement of Work (no fee SOW) and a Data Transfer Agreement (DTA). The DTA protects the privacy of the participants’ data, and the SOW outlines the planned use of the summary statistics. The SOW and DTA will need to be signed by your institution first, and then Viome, before data can be shared. If you are collaborating with investigators at multiple institutions and those institutions must also receive copies of Viome summary statistics, please have a PI from each institution fill out the form to ensure all parties receive access to datasets within a similar timeframe. Finally, please note that each signed SOW and DTA allows use of Viome data only by the signatory institution and its personnel. Each institution that wishes to access or use Viome data must have a signed SOW and DTA covering their access to Viome data. Once a valid SOW and a valid DTA are signed off, Viome will transfer data to the researcher for use in the research project described in the SOW.

## Code availability

For the oral cancer classifier, we have used standard algorithms such as logistic regression to derive the main results in this research. The major parameterization decisions (e.g., L2 regularization) have been described in the “Methods” section. Statistics and Machine-Learning software was written using standard Python libraries such as scikit-learn, pandas, and numpy.
