# Supplementary material for: The salivary metatranscriptome as an accurate diagnostic indicator of oral cancer
Source: NPJ Genom Med. 2021 Dec 8;6:105. doi: 10.1038/s41525-021-00257-x (PMC8654845; doi:10.1038/s41525-021-00257-x)
Supplement: Supplementary file 1 — Supplementary Information [file 41525_2021_257_MOESM1_ESM.pdf]

Supplementary Material

The salivary metatranscriptome as an accurate diagnostic indicator of oral cancer

Guruduth Banavar[1,*], Oyetunji Ogundijo[1], Ryan Toma[1], Sathyapriya Rajagopal[1], Yen Kai Lim[2,3], Kai Tang[2,3], Francine Camacho[1], Pedro J. Torres[1], Stephanie Gline[1], Matthew Parks[1], Liz Kenny[4], Nevenka Dimitrova[5], Ally Perlina[1], Hal Tily[1], Salomon Amar[5], Momchilo Vuyisich[1], Chamindie Punyadeera[2,3,*]

[1] Viome Research Institute, Viome Inc, Bellevue, WA / Los Alamos, NM / New York, NY / San Diego, CA, USA
[2] The Saliva and Liquid Biopsy Translational Laboratory, Institute of Health and Biomedical Innovation, Queensland University of Technology, Brisbane, QLD, Australia
[3] The Translational Research Institute, Woolloongabba, Brisbane, QLD, Australia
[4] The School of Medicine, University of Queensland, Royal Brisbane and Women's Hospital, Brisbane, QLD, Australia
[5] New York Medical College, Valhalla, NY, USA
* Corresponding authors: G.B. guru@viome.com and C.P. chamindie.punyadeera@qut.edu.au

Supplementary Note 1: Clinical Study details

Study inclusion and exclusion criteria. All participants were aged 18 years or older, able to speak and read English, and were willing and able to follow study instructions. We excluded people who were pregnant, had known active infections and/or under antibiotics. All participants were not taking any local and/or systemic antibiotics prior to sample collection at point of diagnosis. For the normal healthy controls, their state of health was assessed using a survey questionnaire on recent history of alcohol or drug abuse or other medical condition; no prior individual history of any cancer (acceptable if family history of cancer); and no previous irradiation to head and neck region.

Clinical labels. Clinical assessments for the Oral Cancer (OC) patients were performed using standard of care biopsies and histopathology evaluations. 45 OC samples were provided TNM codes as specified by [1]. These TNM codes were mapped to 13 Stage I, 16 Stage II, 2 Stage III, and 14 Stage IV samples. The tissue samples were located as shown in Supplementary Figure 1. The OPMD samples captured conditions such as: Epithelial hyperplasia with hyperkeratosis and mild dysplasia, fibroepithelial hyperplasia with hyperkeratosis, mild epithelial dysplasia, mild patchy lichenoid inflammatory change, mild lichenoid dysplasia, lichenoid reaction, hyperplastic squamous mucosa with hyper and parakeratosis, acanthosis associated with lichenoid inflammatory changes, mild non-specific chronic inflammation and overlying parakeratosis, oral lichen planus, and verrucous leukoplakia.

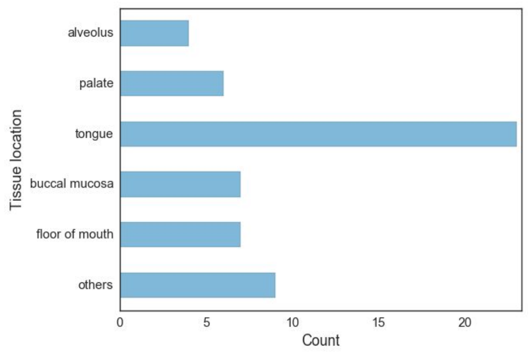

Supplementary Figure 1: Tissue location of OC samples.

Saliva sample collection and processing. Saliva samples were collected from all participants at a resting stage. Participants were asked to refrain from eating and drinking for 1-hour prior to the collection of saliva, with the exemption of drinking plain water to ensure they are fully hydrated. Prior

sample collection, bottled water was given to participants to rinse their mouth. During saliva collection/expectoration, participants sat comfortably in an upright position with the head slightly tilted forward so that saliva pools to the front of the mouth. The participants were asked to pool saliva (head tilted slightly down) in the mouth for about 2-5 minutes and expectorate into a specimen collection cup (at least 1-5 ml of saliva) as per our previous [2]. Collection was done under the supervision / assistance of trained staff. All specimens were preserved using the Viome RNA stabilizer [3], transported back to the laboratory, and stored at -80 °C until further use.

Supplementary Note 2: Lab and bioinformatics details

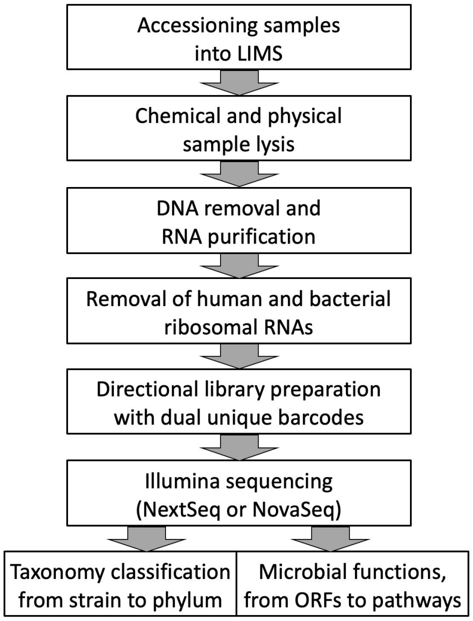

Supplementary Figure 2: Saliva sample processing procedure for NGS workflow

The overall lab and bioinformatic flow is shown in Supplementary Figure 2. For NGS analysis, a saliva specimen is lysed using bead beating in a chemical denaturant; total RNA is extracted from clarified lysate; DNA is removed using DNase; Bacterial and human rRNAs are physically removed from the specimen using a subtractive hybridization method. Biotinylated DNA probes complementary to rRNAs are hybridized to the total RNA and removed using streptavidin magnetic beads. The remaining RNAs are converted into Illumina sequencing libraries. Each specimen is tagged with 11 bp dual unique molecular barcodes; libraries are pooled; the concentration of library pool is determined, and library pools are sequenced on Illumina NovaSeq 6000 to produce sequencing data. The only primers used are the ones that amplify cDNA during the library preparation process. Each primer consists of 1) the standard Illumina clustering sequences, 2) proprietary barcode sequences of 11 nts each and 3) proprietary and custom amplification sequences that amplify the cDNA molecules.

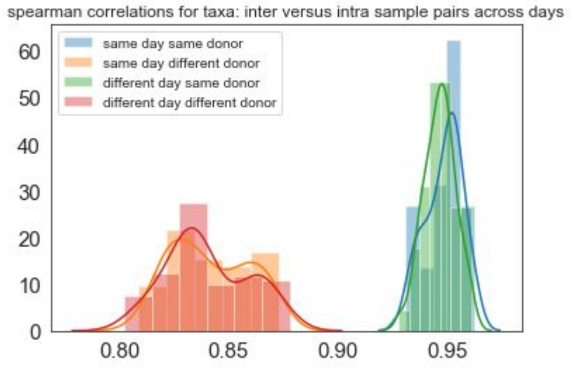

(a)

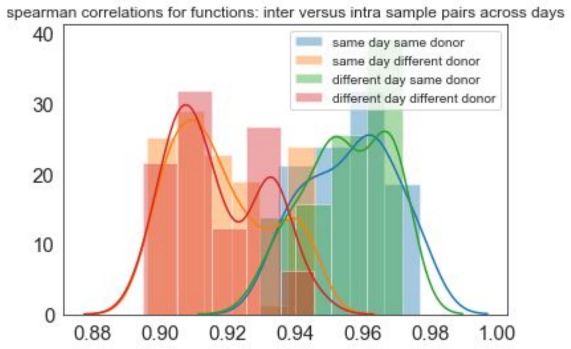

(b)

Supplementary Figure 3: Distribution of Spearman correlations for inter and intra donor saliva sample pairs processed concurrently and independently (a) taxonomy data (b) functional data.

Robustness of lab assay. Supplementary Figure 3 provides a high-level summary of the robustness of our lab process. For this evaluation, we took technical replicates from three saliva donors collected and processed both immediately and stored for 7 days, sequenced in separate batches. We then looked at Spearman correlations between all sample pair combinations

spanning donors, sequencing batches and storage conditions for both active microbial and functional data. We find very high correlations across all sample pairs from the same donor regardless of storage and sequencing batch (see overlap in blue and green distributions in Supplementary Figure 3, mean at 0.96). Additionally, inter-donor sample pairs have lower Spearman correlations which is expected due to biological variation, however, there is no distinction between storage or sequencing batch (see overlap in red and orange distributions in Supplementary Figure 3, mean at 0.91).

Bioinformatics. Once the saliva samples were processed through our lab process and sequenced, we processed the sequenced reads through our bioinformatic pipeline to obtain high-resolution metatranscriptomic data of the oral microbiome. This data includes 1) active microbes identified against Viome's taxonomic catalog, and their relative activities are calculated at three different taxonomic ranks (genus, species, and strain); and 2) active gene-encoded functions, which are functional ortholog assignments (KEGG Orthologs or KOs) annotated for all sequencing reads aligned to the gene catalog (IGC) of the human microbiome and the KEGG databases.

- We downloaded the complete genomes available in NCBI Reference Sequence Database and used the GenBank sequence database for viral genomes.
- Compression of genomes was done during construction of the taxa index. All genomes are compared, and similarities are computed based on those that share the most number of k-mers. Redundancy is handled by iteratively merging sequences into genomes until each of the merged genomes has no sequences >99% identical to any other genomes.
- We used various mock communities composed of a predetermined mixture of microbes (i.e. ATCC ® MSA-2002™ ATCC ® MSA-2003™ and ZymoBIOMICS® Microbial Community Standard) to validate and optimize our bioinformatics pipeline and their thresholds. We defined the minimum hitlength, the approximate number of base pairs of a given read that match the reference genomic sequence with a specific cutoff for mapping.
- The expectation-maximization (EM) algorithm is widely used in computational biology to estimate relative expression levels in the face of read mapping uncertainty arising from multi mapping reads. EM is an iterative algorithm that converges towards the optimal solution for the relative abundance of each taxa. StrainFinder [4] uses the EM algorithm to estimate strain frequencies in complex metagenomic samples.
- We use a very large catalog of compiled microbiome-associated genomes. Open reading frames (ORFs), i.e. protein coding sequences or colloquially "genes", were predicted using common ORF prediction algorithms. Across all genomes, hundreds of millions of genes were identified. Genes were clustered by sequence similarity (homology) into 37 million gene clusters. For each cluster, a single representative gene sequence was chosen.

Summary of metatranscriptomic data. On average, each sample has 1.5 million reads mapping to mRNA. In total, our molecular data consists of 1587 active microbes and 4932 active functions, a total of 6,519 features. On average, each sample has 444 active microbes and 2299 active functional assignments.

Supplementary Note 3: Additional analysis

In addition to using the entire set of features (taxa and KOs) for machine learning purposes as presented in Table 2, we also developed additional machine learning models by separating the taxa & KO features. For Cohort A (discovery cohort), the ROC AUC using taxa features only was 0.85 and using KO features only was 0.84. The ROC AUC for the combined taxa & KO features is the highest at 0.87 and is thus shown in the table. For Cohort C (average-risk OC-only), which has significant overlaps with Cohort A (92 samples from A that were OC-only, plus 7 additional OC patients who were younger than 50 and with no history of tobacco), the ROC AUC for using taxa and KOs separately as features are 0.93 and 0.88, respectively. To err on the conservative side, we only presented in Table 2 the model using both taxa & KO features, which performs at 0.9 ROC AUC (which is between the other two values above).

Supplementary Note 4: Viome Functional Categories

Transcriptomic data support the concept that functional, rather than compositional, properties of oral bacterial communities have more relevance to cancer development. We have built an annotation system that integrates both taxonomic abundances and the functional expression profiles from KOs into higher order biological themes that are relevant to the Oral Cancer phenotype and Oral microbiome in general. We call these biological themes as `Viome Functional Categories (VFC)'. The VFC are unique, highly curated themes that take into account the direction of association of taxa and KO features from the OC predictive model discussed in the paper and provide mechanistic insights into Oral Carcinogenesis. For instance, the functions or pathways resulting in the production or utilization of a specific metabolite like Hydrogen sulfide or carcinogens could be attributed from the curated VFCs. We report a total of 36 VFC that could be grouped into 9 major biological themes relevant to Oral Cancer and Oral microbiome below.

The theme `**ProInflammatory Activities Promoting Carcinogenesis**` provides evidence of a modified polymicrobial synergy and dysbiosis model for bacterial involvement in OC. The following three VFC provide details about the mechanism that induce inflammation and thereby favor carcinogenesis. Here, we report some of the features that are predictive of OC and shed light on some of the mechanisms in oral dysbiosis and periodontal conditions that mediate oral carcinogenesis.

1. Opportunistic Microbial Activities and Oral Pathobionts: The opportunists like "Porphyromonas", "Fusobacterium" and Oral Pathobionts (commensal-derived opportunistic pathogens) such as "Streptococcus sp.", "Gemella sp." are known to mediate oral dysbiosis and lead to subsequent periodontal conditions that might be conducive of OC. These organisms share the ability to attach and invade oral epithelial cells, and communicate with the host epithelium, and ultimately acquire phenotypes associated with cancer such as inhibition of apoptosis, increased proliferation, and increased migration of epithelial cells [5]. Additionally, emerging properties of structured bacterial communities may increase oncogenic potential, and consortia of P. gingivalis and F. nucleatum are synergistically pathogenic within in vivo OC models Among the pathogens positively associated with OC from the model are Porphyromonas, Treponema and Fusobacterium and have higher abundances in oral swabs of patients with oral cancer. [6].
2. LPS Production Activities: Bacterial outer membrane lipopolysaccharides are entities that mediate proinflammatory immune response and inflammation host cells. LPS regulates gene expression of pro-inflammatory cytokines through activation of toll-like receptor 4 (TLR4) via NF-kB [7]. The 'O antigens', an extremely polymorphic polysaccharide binds to LipidA to form the LPS outer-membrane of Gram-negative bacteria thereby imparting antigenic specificity to the organism. For instance, LPS from Porphyromonas, a positively associated taxa from the OC model, is known to activate macrophages and increase NO production of cancer cell lines [8]. Furthermore, a functional KO implied in LPS production is positively associated from the OC model.
3. Biofilm and Virulence Pathways: The OC model predicts a number of functional features associated with bacterial virulence promoting inflammation and positively associated with OC. For instance, sugar transport and chemotaxis associated KOs from oral microbes that are deterministic of virulence and pathogenesis [9] are predicted. Many lytic enzymes, cell wall synthesis associated transporter and phospholipase are the other virulence determining functional KOs that are found as predictive of OC from the model.

AntiInflammatory and Antimicrobial Pathways: The commensal bacteria Streptococcus sp. establishes in the human oral cavity a few hours after birth and remains there as a predominant commensal and as a primary colonizer of biofilms. Upon strong adhesion mediated by the glycosylated surface-exposed proteins Streptococcus sp. promotes innate immunity by suppressing proinflammatory cascades as well as by producing anti-microbial substances like bacteriocins that antagonizes the virulent streptococci involved in tooth decay or pharyngitis or pathogens involved in periodontitis [10]. Similarly, Streptococcus sp. 2, also an early colonial member of oral biofilm produces H2O2 to inhibit the growth of competitors, like the mutans streptococci, as well as strict anaerobic middle and later colonizers of the dental biofilm. Interestingly, Veillonella species, possess a putative catalase gene that mediates resistance to the Streptococcus sp.2 thereby enabling direct physical interaction (co-aggregate) with

Streptococcus sp.2 as well as Fusobacterium sp. that are late colonizers of biofilm [11]. It is interesting to note that Fusobacterium is a positive predictor of OC while Streptococcus sp.1 is negatively associated. Furthermore, the model captures functional determinant of antimicrobial resistance gene and catalase as positive predictors of OC.

Hydrogen sulfide (H2S), a gaseous transmitter, is associated with oral periodontitis and is one of the main causes of halitosis and is generally associated with many oral diseases including OC [12]. Hydrogen sulfide production pathways including enzymes that produce H2S are increased in different human malignancies. The expression of both enzymes and cellular H2S levels increase tumor survival and promote tumor dedifferentiation [13]. Among the taxa, members of the Streptococcus group, Fusobacterium and Porphyromonas, some of the known producers of oral H2S are in turn also predicted from the model to be cancer specific. The model predicts three H2S producing KOs are also positively associated with OC.

Cancer-Specific Energy Metabolism and Utilization: In cancer cells, the Pentose Phosphate Pathway (PPP) together with glycolysis, coordinates glucose flux and supports the cellular biogenesis of macromolecules such as lipids and DNA for energy production. An increased PPP flux in human cancer cells is indicative of its role in meeting the bioenergetic demands of cancer cell proliferation and contribution to the Warburg effect [14]. Enzymes involved in pentose interconversion, as well in pentose-5P production, are positively associated features from the model suggest microbial dysregulation of PPP flux in human cancer cells.

Lack of Protective or Detox mechanisms: Detoxification mechanisms are essential for multitude of cellular processes, including cell differentiation, proliferation, and apoptosis, and disturbances in their homeostasis are implicated in the etiology and/or progression of a number of human diseases, including cancer, diseases of aging, inflammatory, immune, metabolic, and neurodegenerative diseases With the advent of cancer, a number of protective and detoxifying mechanisms are dysregulated in the cell in response to combat intracellular and extracellular stress. From the model, we see an upregulation of thiol based deconjugation functions, to be positively associated with cancer. Low Molecular weight (LMV) thiols are produced by gram-positive firmicutes that function in protecting cells against reactive oxygen species (ROS) and reactive electrophilic species, antibiotics, alkylating agents, as well as heavy metals [15]. On the other hand, microbial glutathione mediated stress response is negatively associated in the model. Thus, a preferential microbial thiol-based detoxification of ROS and reactive electrophilic species is known to be associated with OC from our model. Along with these, the antibacterial as well as AntiInflammatory functions such as catalase and butyrate production are downregulated and are found to be negatively associated with cancer.

Protein fermentation as a tumorigenic mechanism: Protein fermentation results in the accumulation of by-products that are resourceful for the cancer cells hence is a favorable environment as a tumor promoting microenvironment. Polyamines such as putrescine, cadaverine and spermidine are products of microbial protein fermentation are essential for normal cell growth, and their depletion results in cytostasis. Polyamine metabolism is frequently dysregulated in cancer and elevated polyamine levels are necessary for transformation and tumor progression [16]. For instance, the spermidine is needed as a precursor of hypusine (a post-translational addition to eukaryotic initiation factor 5A isoform 1 (eIF5A) that is necessary to prevent ribosomal stalling in the translation of mRNAs encoding polyproline tracts and certain other amino acid combinations. The MYC oncogene plays a role in hypusine formation by driving the transcription of the gene encoding ornithine decarboxylase (ODC) and indirectly increasing the availability of spermidine for hypusine synthesis [17-18]. A deoxyhypusine synthase requiring spermidine is identified as a positively associated feature from the model. The cancer cells tend to accumulate increased concentrations of polyamines through increased uptake via their Polyamine Transport System (PTS) [19]. With increased microbial protein breakdown, cadaverine transport systems transport cadaverine into the host cell and promote carcinogenesis and such a polyamine antiporter is identified as positively associated with cancer from the model. The cellular protein degradation produces ammonia as a by-product which is recycled into central amino acid metabolism to maximize nitrogen utilization [20]. Increased microbial ammonia production is noted from KOs such as glutamate dehydrogenase associated with OC from the model.

Benzaldehyde, arsenite, and other carcinogenic toxins: The exposure to synthetic chemicals such as dyes, organopesticides and pharmaceuticals increases the toxicity burden of cells that elevates the cancer-causing potential in general. A feature that contributes to the production of benzaldehyde is detected as the top second feature from the predictive model of OC. Benzaldehyde is a potential

biomarker for OC in breath test [21]. Further, traces of fluorobenzoate metabolism and acetaldehyde production KOs are also observed to be predictive of oral cancer. Exposure to metallic arsenic is toxic to the cells and the extent of arsenic toxicity is dependent on its oxidative state [22-23]. Arsenite transporters are positive predictors of OC from the model.

While the above tumor promoting functions are all positively associated with the OC, a host of taxa and related activities are also detected as predictive of cancer. These include the **Skin and genital microbes** and several pathway functions such as Inorganic Ion Transport Pathways, Amino acid production and Vitamin Biosynthesis pathways and Cofactor and coenzyme synthesis. Amongst the most prominent negative associated features include the Oral Commensal and plaque microbes such as Streptococcus as well as several pathways such as Energy production, Cell wall biosynthesis and sporulation, Antibiotic resistance, Microbial heat and osmolarity mediated stress which are related to Common oral microbiome related functions not necessarily implicated in cancer. Several pathways such as Cell cycle and DNA repair and Carbohydrate metabolism and transport pathways are found to be less predictive as the features are found to be predictive of both cancer as well as controls.

Supplementary References

1. The 8th edition of the American Joint Committee on Cancer/Union for International Cancer Control (AJCC/UICC) tumour-node-metastasis (TNM) staging system. https://www.facs.org/Quality-Programs/Cancer/AJCC (2019) .
2. Lim, Y.K. & Punyadeera, C. A pilot study to investigate the feasibility of transporting saliva samples at room temperature with MAWI Cell Stabilization buffer. *Cogent Biology* **4.** 1470895 (2018).
3. Hatch, A. et al. A robust metatranscriptomic technology for population-scale studies of diet, gut microbiome, and human health. *International Journal of Genomics* **2019.** (2019).
4. Smillie, C.S. et al. Strain Tracking Reveals the Determinants of Bacterial Engraftment in the Human Gut Following Fecal Microbiota Transplantation. *Cell Host Microbe* **23.** 229-240 (2018).
5. Karpiński, T.M. Role of Oral Microbiota in Cancer Development. *Microorganisms* **7.** 20 (2019).
6. Chattopadhyay, I., Mukesh, V., & Madhusmita, P. Role of Oral Microbiome Signatures in Diagnosis and Prognosis of Oral Cancer. *Technology in Cancer Research & Treatment* **18.** (2019).
7. Goncalves, M. et al. Effect of LPS on the Viability and Proliferation of Human Oral and Esophageal Cancer Cell Lines. *Braz. arch. biol. technol* **59.** e16150485 (2016).
8. Utispan, K., Pugdee, K., & Koontongkaew, S. Porphyromonas gingivalis lipopolysaccharide-induced macrophages modulate proliferation and invasion of head and neck cancer cell lines. *Biomedicine & Pharmacotherapy* **101.** 988-995 (2018).
9. Matilla, M.A. & Krell, T. The effect of bacterial chemotaxis on host infection and pathogenicity. *FEMS Microbiology Reviews* **42.** fux052 (2018).
10. Kaci, G. et al. Anti-Inflammatory Properties of Streptococcus Salivarius, a Commensal Bacterium of the Oral Cavity and Digestive Tract. *Applied and Environmental Microbiology* **80.** 928–34 (2014).
11. Zhou, P., Li, X., I., Huang, I., & Qi, F. Veillonella Catalase Protects the Growth of Fusobacterium Nucleatum in Microaerophilic and Streptococcus Gordonii-Resident Environments. *Applied and Environmental Microbiology* **83.** (2017).
12. Zhang, S. et al. Hydrogen Sulfide Promotes Cell Proliferation of Oral Cancer through Activation of the COX2/AKT/ERK1/2 Axis. *Oncology Reports* **35.** 2825–32 (2016).
13. Patel, S. et al. Increased Nicotinamide Phosphoribosyltransferase and Cystathionine-Beta-Synthase in Oral Cavity Squamous Cell Carcinomas. *Int J Clin Exp Pathol* **10.** 702-707 (2017).
14. Lu, J., Ming, T., & Qingsong, C. The Warburg Effect in Tumor Progression: Mitochondrial Oxidative Metabolism as an Anti-Metastasis Mechanism. *Cancer Letters* **356.** 156-64 (2015).
15. Chandrangsu, P., Loi, V., Antelmann, H., & Helmann, J. The role of bacillithiol in Gram-positive Firmicutes. *Antioxidants \& redox signaling* **28.** 445-462 (2018).
16. Murray-Stewart, T.R., Woster, P.M. & Casero Jr, R.A. Targeting polyamine metabolism for cancer therapy and prevention. *Biochemical Journal* **473.** 2937-2953 (2016).
17. Park, M.H., Nishimura, K. Zanelli, C.F & Valentini, S.R. Functional significance of eIF5A and its hypusine modification in eukaryotes. *Amino acids* **38.** 491-500 (2010).
18. Casero, R.A., Stewart, T.M. & Pegg, A.E. Polyamine metabolism and cancer: treatments, challenges and opportunities. Nature Reviews Cancer 18. 681-695 (2018).
19. Palmer, A.J., Ghani, R.A., Kaur, N., Phanstiel, O. & Wallace, H.M. A putrescine–anthracene conjugate: a paradigm for selective drug delivery. *Biochem J* **424.** 431-438 (2009).
20. Moreno-Sánchez, R. et al. Physiological Role of Glutamate Dehydrogenase in Cancer Cells. *Frontiers in Oncology* **10.** 429 (2020).
21. Bouza, M., Gonzalez-Soto, J., Pereiro R., de Vicente, J.C. & Sanz-Medel, A. Exhaled breath and oral cavity VOCs as potential biomarkers in oral cancer patients. *Journal of Breath Research* **11.** 016015 (2017).
22. Hughes, M.F. Arsenic toxicity and potential mechanisms of action. *Toxicology Letters* **133.** 1-16 (2002).
23. Chen, C., Kuo, T. & Wu, M. Arsenic and cancers. *The Lancet* **331.** 414-415 (1988).
