# Supplementary material for: The salivary metatranscriptome as an accurate diagnostic indicator of oral cancer
Source: NPJ Genom Med. 2021 Dec 8;6:105. doi: 10.1038/s41525-021-00257-x (PMC8654845; doi:10.1038/s41525-021-00257-x)
Supplement: Supplementary file 2 — Reporting Summary [file 41525_2021_257_MOESM2_ESM.pdf]

nature research

Corresponding author(s): Guruduth Banavar (guru@viome.com)

Last updated by author(s): Nov 16, 2020

Reporting Summary

Nature Research wishes to improve the reproducibility of the work that we publish. This form provides structure for consistency and transparency in reporting. For further information on Nature Research policies, see our Editorial Policies and the Editorial Policy Checklist.

Statistics

For all statistical analyses, confirm that the following items are present in the figure legend, table legend, main text, or Methods section.

n/a	Confirmed	
☐	☒	The exact sample size (*n*) for each experimental group/condition, given as a discrete number and unit of measurement
☐	☒	A statement on whether measurements were taken from distinct samples or whether the same sample was measured repeatedly
☐	☒	The statistical test(s) used AND whether they are one- or two-sided<br>*Only common tests should be described solely by name; describe more complex techniques in the Methods section.*
☐	☒	A description of all covariates tested
☐	☒	A description of any assumptions or corrections, such as tests of normality and adjustment for multiple comparisons
☐	☒	A full description of the statistical parameters including central tendency (e.g. means) or other basic estimates (e.g. regression coefficient) AND variation (e.g. standard deviation) or associated estimates of uncertainty (e.g. confidence intervals)
☐	☒	For null hypothesis testing, the test statistic (e.g. *F*, *t*, *r*) with confidence intervals, effect sizes, degrees of freedom and *P* value noted<br>*Give P values as exact values whenever suitable.*
☒	☐	For Bayesian analysis, information on the choice of priors and Markov chain Monte Carlo settings
☒	☐	For hierarchical and complex designs, identification of the appropriate level for tests and full reporting of outcomes
☒	☐	Estimates of effect sizes (e.g. Cohen's *d*, Pearson's *r*), indicating how they were calculated

Our web collection on statistics for biologists contains articles on many of the points above.

Software and code

Policy information about availability of computer code

Data collection	No software was used to collect the data in this study
Data analysis	Bioinformatics methods are described in the paper. Statistics and Machine Learning software was written using standard Python libraries such as scikit-learn, pandas, and numpy.

For manuscripts utilizing custom algorithms or software that are central to the research but not yet described in published literature, software must be made available to editors and reviewers. We strongly encourage code deposition in a community repository (e.g. GitHub). See the Nature Research guidelines for submitting code & software for further information.

Data

Policy information about availability of data

All manuscripts must include a data availability statement. This statement should provide the following information, where applicable:

- Accession codes, unique identifiers, or web links for publicly available datasets
- A list of figures that have associated raw data
- A description of any restrictions on data availability

The dataset for the discovery cohort (Cohort A) is provided for download at http://dx.doi.org/10.6084/m9.figshare.13244243

Field-specific reporting

Please select the one below that is the best fit for your research. If you are not sure, read the appropriate sections before making your selection.

☒ Life sciences ☐ Behavioural & social sciences ☐ Ecological, evolutionary & environmental sciences

For a reference copy of the document with all sections, see nature.com/documents/nr-reporting-summary-flat.pdf

Life sciences study design

All studies must disclose on these points even when the disclosure is negative.

Sample size	This was a pilot study with the goal of exploring the signal that could be detected in a dataset of N=433 samples organized into multiple cohorts corresponding to the clinical requirements of oral cancer.
Data exclusions	Data exclusions were based on pre-established criteria for the quality of data, including RNA concentration, number of taxa and/or KOs, etc. We excluded 1 (one) sample based on these criteria.
Replication	Cross-validation across N-1 iterations of the training set was intended to ensure replication of the results.
Randomization	We could not randomize subjects. However, we performed error analysis of different groups within covariates, and ensured that these assignments did not influence the prediction of our model.
Blinding	This is an observational study (not an interventional study), therefore blinding was not applicable.

Reporting for specific materials, systems and methods

We require information from authors about some types of materials, experimental systems and methods used in many studies. Here, indicate whether each material, system or method listed is relevant to your study. If you are not sure if a list item applies to your research, read the appropriate section before selecting a response.

Materials & experimental systems

n/a	Involved in the study	
☒	☐	Antibodies
☒	☐	Eukaryotic cell lines
☒	☐	Palaeontology and archaeology
☒	☐	Animals and other organisms
☐	☒	Human research participants
☐	☒	Clinical data
☒	☐	Dual use research of concern

Methods

n/a	Involved in the study	
☒	☐	ChIP-seq
☒	☐	Flow cytometry
☒	☐	MRI-based neuroimaging

Human research participants

Policy information about studies involving human research participants

Population characteristics	Age, sex, and medical diagnosis of oral cancer are described in Table 1 of the manuscript.
Recruitment	Recruitment of oral cancer patients was performed by hospital staff. Cancer-free subjects were recruited from the general population.
Ethics oversight	This study was approved by the Queensland University of Technology and University of Queensland Medical Ethical Institutional Boards (HREC no.: 1400000617 and HREC no.: 2017000662 respectively) and the Royal Brisbane and Women's Hospital (HREC no.: HREC/12/QPAH/381) Ethics Review Board. Written informed consent was obtained from all participants and all of the methods in this study were performed in accordance with the relevant guidelines and regulations.

Note that full information on the approval of the study protocol must also be provided in the manuscript.

Clinical data

Policy information about clinical studies

All manuscripts should comply with the ICMJE guidelines for publication of clinical research and a completed CONSORT checklist must be included with all submissions.

Clinical trial registration	We have not registered this pre-pilot study yet, but plan to register the larger pilot study required for an FDA submission.

Study protocol	Study protocol is available by emailing to guru@viome.com
Data collection	Samples and data were collected by C. Punyadeera under the auspices of Queensland University of Technology
Outcomes	Primary outcome measure was the cancer diagnosis based on histopathology reports
